# A labor requirements function for sizing the health workforce

**DOI:** 10.1186/s12960-018-0334-4

**Published:** 2018-12-04

**Authors:** Sofia Cruz-Gomes, Mário Amorim-Lopes, Bernardo Almada-Lobo

**Affiliations:** 1 INESC TEC and Faculty of Engineering, University of Porto, Porto, Portugal; 2 Católica Porto Business School, Porto, Portugal

**Keywords:** Health Human Resources, Labor requirements function, Labor productivity, Opportunity costs

## Abstract

**Background:**

Ensuring healthcare delivery is dependent both on the prediction of the future demand for healthcare services and on the estimation and planning for the Health Human Resources needed to properly deliver these services. Although the Health Human Resources planning is a fascinating and widely researched topic, and despite the number of methodologies that have been used, no consensus on the best way of planning the future workforce requirements has been reported in the literature. This paper aims to contribute to the extension and diversity of the range of available methods to forecast the demand for Health Human Resources and assist in tackling the challenge of translating healthcare services to workforce requirements.

**Methods:**

A method to empirically quantify the relation between healthcare services and Health Human Resources requirements is proposed. For each one of the three groups of specialties identified—Surgical specialties, Medical specialties and Diagnostic specialties (e.g., pathologists)—a Labor Requirements Function relating the number of physicians with a set of specialty-specific workload and capital variables is developed. This approach, which assumes that health managers and decision-makers control the labor levels more easily than they control the amount of healthcare services demanded, is then applied to a panel dataset comprising information on 142 public hospitals, during a 12-year period.

**Results:**

This method provides interesting insights on healthcare services delivery: the number of physicians required to meet expected variations in the demand for healthcare, the effect of the technological progress on healthcare services delivery, the time spent on each type of care, the impact of Human Resources concentration on productivity, and the possible resource allocations given the opportunity cost of the physicians’ labor.

**Conclusions:**

The empirical method proposed is simple and flexible and produces statistically strong models to estimate Health Human Resources requirements. Moreover, it can enable a more informed allocation of the available resources and help to achieve a more efficient delivery of healthcare services.

**Electronic supplementary material:**

The online version of this article (10.1186/s12960-018-0334-4) contains supplementary material, which is available to authorized users.

## Background

Ideally, a health system provides quality and timely care services, contributing to a healthy population. However, most health systems have been facing rising care volumes and health expenditures, which is a significant problem considering that resources are scarce. As human resources usually represent the largest item in the healthcare budget, and since no health system can deliver healthcare services without them, Health Human Resources (HHR) are widely recognized as the most important input of a health system [1].

Planning the healthcare workforce has long been a major challenge for health policy-makers. Although the widely recognized relevance of HHR planning is not new, attention and resources allocated to workforce planning have increased in recent years [2]. Imbalances in the health workforce (disequilibrium between the demand for and the supply of health professionals) are becoming a major concern for both developed and developing countries [3, 4], increasing the need to achieve a balance between the available and the required workforce to provide healthcare services [5].

HHR planning comprises the study and analysis of four key elements: supply, demand, potential gap, and possible solutions to solve imbalances [6, 7]. Typically, most HHR planning studies address the supply side, the demand side, or a combination of both. Studies focusing on the supply side aim to forecast the HHR that will exist in the future, analyzing factors that influence the movement of professionals into, through and out of the healthcare workforce, as well as their motivations and organization [8]. On the other hand, studies approaching the demand side aim to predict the HHR that will be needed or demanded in the future. These studies focus on the evolution of factors driving the demand for health services and on the estimation of the HHR required to deliver these services [9]. Perhaps due to the relative simplicity of the data required to address the supply side, considerable attention has been placed on supply approaches, with fewer endeavors on the demand side [10]. In fact, most specialty workforce studies limited their scope of analysis to the supply side, with only about 20% addressing the demand [11].

On the demand side, four main approaches to project future HHR requirements can be identified in the literature: health needs, service utilization/demand, service targets, and workforce-to-population ratios [2, 5, 8, 12–14]. These approaches differ in the way the required healthcare services are identified: approaches based on health needs use epidemiological information to estimate the effect of diseases’ incidence and prevalence in the future demand for healthcare services; approaches based on demand consider the current levels of services utilization, assuming a constant relation between demand and its drivers, with only the drivers’ level changing over time; service-target approaches aim to identify future needs by establishing targets to the production and delivery of healthcare; and population-ratio approaches estimate future HHR requirements using demographic projections and desired health worker-to-population ratios that can be derived from benchmarks, studies, or deliberations of policy-makers [15]. Of these four approaches, only the worker-to-population ratio estimates directly the health workforce requirements, while the other three require the conversion of healthcare services into workforce needs [14].

Although planning HHR is a fascinating and widely researched topic, and despite the number of methodologies that have been used, no consensus has been reached in the scientific literature on the best way of planning for future HHR. In fact, while there is a growing agreement that planning the demand for HHR should be based on healthcare needs [7], less accordance exists on the best way to translate needs in HHR requirements.

Several methods of converting services into workforce requirements can be found in the literature. The most commonly used consists of assessing the time required to complete tasks, measured by direct observation (time-motion studies, activity sampling techniques, and patient-flow analysis) or using expert’s opinion [16–18]. This method is not very demanding in terms of data, but defining the necessary tasks can be difficult and time-consuming. Moreover, there is the risk of over-estimating the HHR requirements [15]. Another method, based on productivity patterns, consists in applying labor productivity measures to the expected demand for healthcare. This approach can either assume the maintenance of the actual productivity levels or use an ideal or desirable productivity level [17, 19]. More recently, microsimulation models are also being applied to forecast the health workforce. These models have the advantage of offering information with a high level of granularity [20], but they usually apply specific staffing ratios to the health services delivered, which are assumed to remain constant over the time [21]. Involving economic fundamentals in HHR planning has also become popular. In this context, the estimation of production functions (PF) relating healthcare inputs (HHR and capital in health facilities) to healthcare outputs (delivered services) has become a widely used method in HHR planning [22, 23]. Although the economic concepts and assumptions underlying the use of PF are suitable to the problem [24], the complexity of handling multiple outputs contributed to a rising interest in the creation of indexes combining different outputs [25] and in nonparametric approaches for the estimation of production frontiers, such as data envelopment analysis [26].

An inverse PF can also be used to model this production process [27]. This function, commonly known as input requirements function (IRF), is another way of overcoming the limitation of handling multiple outputs [28], keeping the same key economic concepts. Employing IRFs to understand the demand for labor became a very popular approach in many sectors, including banking [29], manufacturing [30], and agriculture [31], since labor can be sized according to needs and capacity. Despite the strengths and adequacy of using IRF and its popularity in other fields, the use of IRF in the healthcare literature is almost inexistent. In fact, to the best of our knowledge only one study applied an IRF in HHR planning context: Lipscomb et al. [28] applied a Cobb-Douglas IRF to cross-sectional data on US Veterans Affairs hospitals, relating the number of physicians with workload variables, number of residents, and size of the hospital. This study presents two main limitations: (i) the inflexible functional form selected for the IRF and (ii) not to consider—due to the empirical data used in the analysis—a time component to capture the technological changes and the productivity effects on the delivery of healthcare, and consequently on the physicians’ requirements. Other health-related studies applied an IRF, but with different purposes: Kumbhakar [30] used an IRF to estimate the minimum per capita health expenditures required to attain a particular level of Disability Adjusted Life Expectancy using panel data on World Health Organization member countries, and Gunning and Sickles [32] used a generalized Leontief IRF to perform a multi-product cost analysis, based on data from American private practices.

As such, the work hereby presented aims to introduce a method to describe and quantify the relation between healthcare services and workforce requirements, assisting on the challenging task of translating health services to HHR, in this way making a contribution to the extension and diversity of the available methods to forecast the demand for HHR. Our approach differs from previous studies by conjoining three main aspects. First, we consider a flexible IRF to model the relation between healthcare services and human resources, a specification based on the assumption that health managers and decision-makers can size and adjust the workforce levels in response to the expected demand. Second, we analyze hospital care by specialty. Although hospital care has been broadly researched, surprisingly not enough attention has been given to specialty-specific approaches, which have the advantage of capturing relevant specificities that otherwise pass unnoticed. Finally, we use panel-data when previous works consider a purely cross-sectional approach. Our approach may improve the statistical reliability of the results and allows for the analysis of the labor productivity and its impact on the healthcare services delivered over time.

The proposed method is meant to be used as a tool to estimate the number of physicians required to meet expected variations in the demand for healthcare. Additionally, it is our goal to contribute to a deeper knowledge on healthcare delivery process, on the possible options of HHR allocation and on the technological progress in healthcare delivery, as they may be relevant drivers for the future needs of HHR.

## Methods

### Data

We use data from the Annual Survey of Hospitals conducted by the Portuguese Institute of Statistics (INE). This data is collected through a mandatory survey, and it reports hospitals’ activity and resources. From the range of hospitals reporting data, we focused on public official hospitals, excluding private and non-public official hospitals, for which access is not universal (military, paramilitary, and prisons hospitals).

We constructed a panel dataset comprising information on 142 hospitals during a 12-year period by compiling information from the surveys between 1999 and 2010. The period after 2010 was not considered due to a change in coding: since 2011 the hospitals’ unique identifier changed and the mapping between the two identification codes (the one used until 2010 and the one used afterwards) is unknown, which creates a discontinuity and invalidates a joint panel analysis for the periods before and after 2011. Moreover, due to recent changes that occurred in the Portuguese Health System some hospitals were closed, created, or merged during the considered period. Our unbalanced panel includes all the hospitals with reported activity for at least one of the years in analysis, even if they were no longer active at the end of the period. The inclusion of hospitals that were not in activity throughout the whole period was meant to eliminate the survivorship bias.

The final sample is composed of 1236 observations of hospitals’ capacity and services utilization by medical specialty.

### Analysis

The heart of our analysis is an input requirements function. The IRF, first introduced by Diewert [27], is an inverse production function (PF): while the PF gives the maximum amount of output that can be produced with a given amount of inputs, the IRF gives the minimum amount of an input that is required to attain a certain level of outputs, holding the other inputs constant. Thus, an IRF can take as dependent variable any of the inputs used in the production, which are usually divided in labor (variable inputs) and capital (fixed inputs).

Since the main purpose of our study is to provide a method to estimate the workforce needed to deliver healthcare services, we consider a labor requirements function (LRF), a specification meant to derive the minimum amount of labor that is required to produce a given level and mix of outputs, with a given level of capital. Indeed, the LRF form seems more suited to the healthcare field than the traditional PF: while the PF specification assumes the amount of labor as exogenous and the outputs as endogenous, the LRF implicitly assumes that workforce levels may be determined and changed according to a given expected demand for healthcare services.

The production of healthcare services can generally be represented by:

1 $$ Y=f\left(L,K\right), $$

where *f* denotes the production technology and *Y*, *L*, and *K* are the vectors of healthcare outputs, labor, and capital variables, respectively.

The above relation can be transformed into an LRF as follows:

2 $$ L=f\left(Y,K\right). $$

Of the parametric functional forms that can be used to model the healthcare services production, the traditional Cobb-Douglas and the transcendental logarithmic (translog) forms are the most popular [33]. We assumed a translog form for its flexibility and for exhibiting several interesting benefits: it is a generalization of the Cobb-Douglas function that imposes less restrictions on the elasticities, and despite assuming a nonlinear relationship between output and inputs, it provides a second-order Taylor’s approximation that can be estimated using linear models.

Assuming that *n* outputs can be produced using labor (*L*) and *m* capital inputs (*K*), the translog LRF can then be written as follows:

3 $$ \ln (L)={\propto}_0+\sum \limits_{i=1}^n{\propto}_i\ln \left({Y}_i\right)+\frac{1}{2}\sum \limits_{i=1}^n\sum \limits_{j=1}^n{\propto}_{ij}\ln \left({Y}_i\right)\ln \left({Y}_j\right)+\sum \limits_{k=1}^m{\beta}_k\ \ln \left({K}_k\right)+\frac{1}{2}\sum \limits_{k=1}^m\sum \limits_{l=1}^m{\beta}_{kl}\ \ln \left({K}_k\right)\ln \left({K}_l\right)+\frac{1}{2}\sum \limits_{i=1}^n\sum \limits_{k=1}^m{\gamma}_{ik}\ln \left({Y}_i\right)\ln \left({K}_k\right)+\mu, $$

where *L* is the labor variable, *Y*_*i*_ the output variable *i*, *K*_*k*_ the capital variable *k*, ∝_0_ represents the constant term, ∝_*i*_ and *β*_*k*_ stand for the coefficients of output *i* and capital variable *k*, ∝_*ij*_, *β*_*kl*_ and *γ*_*ik*_ represent the parameters for the second-order terms, and *μ* represents the error term of the model.

The logarithmic transformation reduces the distributions’ asymmetry and makes the statistical inference more intuitive, as the results are in the form of elasticities. Nonetheless, the use of logarithms carries the difficulty of handling observations with null values. We opted for retaining null-valued observations by substituting them for a very low positive value (0.00001), an approach typically used to overcome this issue.

We identified three main types of specialties according to the healthcare services produced: medical, surgical, and diagnostic-related. Considering the differences between the specialties, we propose a different LRF model, based on Eq. (3), for each type of specialty (Eq. A.1, A.2 and A.3 in the Additional file 1). The main difference between the three models lies on the variables representing the outputs, which were selected according to the activities performed by each type of specialty. Medical specialties are the ones providing inpatient and outpatient care, while surgical specialties carry out the same procedures as medical specialties but also perform surgeries, and diagnostic specialties perform diagnostic and therapeutic procedures.

The public health sector has several objectives, including providing timely treatment to patients, assuring quality of the delivered care, and meeting budget and resource constraints. However, as profit maximization is not an explicit goal, inputs and outputs are not jointly selected. Therefore, a single equation estimation perfectly fits the problem.

Additionally, understanding HHR productivity and technological progress in healthcare delivery is also of major interest, as their evolution may be relevant drivers of future HHR needs. Thus, we propose the estimation of a single LRF using period fixed effects (FE). The selection of a fixed effects (FE) model, also known as least squares dummy variable (LSDV), was due to its ability to capture the effects that are specific to the time period and common to all the entities (hospitals). Thus, these effects can be interpreted as the impact of technological change and labor productivity over the time. Also, we use robust estimators of the variance-covariance matrix to guarantee efficient estimators and ensure valid statistical inference, since the structure of our models may exhibit both heteroscedasticity and serial correlation.

Moreover, we perform two statistical tests: one to assess the adequacy of considering period FE in the models and another to verify the adequacy of the functional form selected for the models (translog). In order to assess the adequacy of the FE estimation, we test the FE redundancy (a test on the joint significance of the fixed effects parameters). Through the rejection of the null hypothesis, it is possible to conclude that the period fixed effects are not redundant to the model and should be considered. Moreover, we perform a test on the joint significance of the second-order terms of the models to infer if the translog form is a good choice for the functional form of our models. In a similar fashion to the previous test, the rejection of the null hypothesis indicates that the second-order terms of the models are not redundant, which implies that the translog form should be used instead of the traditional Cobb-Douglas function.

Based on the FE estimates, we can infer about the technological change rates and the evolution of physicians’ productivity over the considered period, quantify the relation between healthcare services delivery and number of physicians, and understand changes on the process of delivering healthcare over time.

The estimates for the LRF parameters do not have such a clear meaning. However, other useful insights arise from further analysis of these estimates. Elasticities of mean-labor use, which give the labor changes needed to meet a given variation in the production levels, are calculated (Eq. A.4 in the Additional file 1) for each healthcare output using the mean-values of the explanatory variables (Additional file 1: Table S1). In turn, these elasticities are used to calculate the marginal rates of technical substitution (MRTS) of labor between the different healthcare services (Eq. A.5 in the Additional file 1) and the returns to scale (RTS) of labor (Eq. A.6 in the Additional file 1).

In our context, the MRTS is the rate at which one healthcare service can be substituted for another without changing the level of labor, which can be interpreted as the opportunity cost of physicians’ time between the different healthcare services. The RTS represent a measure of the labor changes that are required to meet a variation of all the healthcare outputs by the same proportion, which is useful to inform about the effect of changes in the scale of labor use on productivity.

### Selected variables

Variables that represent the inputs to produce healthcare services (labor and capital), the healthcare outputs, and eventually other factors influencing the production of healthcare need to be selected for applying the LRF model.

When compared to other developed countries, healthcare in Portugal is very physician-intensive. In fact, Portugal has a relatively high ratio of physicians per 1000 population (4.6 in 2017, when the average in the OECD countries was 3.4) and a low ratio of nurses (6.3 nurses per 1000 population vs. 9.0 in the OECD countries). Additionally, and because ensuring the adequate number of physicians requires a timely planning—due to the long educational path for becoming a physician—our focus is on physicians’ requirements. Hence, labor (the dependent variable) is represented only by the number of physicians. To factor in the stock of capital, we use the number of rooms, a proxy to the fixed inputs available to all the production lines, including facilities that are used for outpatient care (rooms for consultations), inpatient care (wards), and surgeries (operating rooms). Finally, the main production lines are modeled as production outputs: inpatient care, measured by the number of inpatient discharges; outpatient care, measured by the number of outpatient visits; surgeries, measured by the number of procedures; and diagnostic and therapeutic procedures, also measured by the number of procedures. Except for the variable used as a proxy for capital, all the others are specialty-specific.

Other factors capable of influencing the process of healthcare production and physicians’ productivity can also be included in the model, such as the number of nurses, residents, and other health-staff working in hospitals, or additional variables related to other fixed inputs, such as the number of beds. However, a correlation analysis (Additional file 1: Table S2) shows these variables to be redundant, as they are extremely correlated to the other variables already factored in in the model (e.g., high correlation between the number of nurses and the size of the hospital, measured by the number of rooms, or between the number of beds and the number of inpatient discharges). Therefore, in order to avoid multicollinearity problems, such as imprecise estimates and invalid statistical inference, we omit these variables from the estimated models without any prejudice to the generality of the results. However, we also present the original LRF models for each type of specialty, which can be used in contexts where these variables are not that correlated (Eq. A.7, A.8 and A.9 in the Additional file 1).

Of the 52 specialties, we selected three for the statistical analysis, one from each specialty group: general surgery (surgical), internal medicine (medical) and anatomical pathology (diagnostic).

## Results

### Descriptive statistics

The descriptive statistics of the variables included in each model are presented in Table 1. The general surgery LRF estimation uses 956 observations, comprising 112 different hospitals between 1999 and 2010. Portuguese hospitals have, on average, 13 general surgeons, each one producing around 700 outpatient visits, 145 inpatient discharges, and 140 surgeries per year. The sample used on the internal medicine LRF estimation includes 1014 observations from 128 hospitals. Portuguese hospitals with an Internal Medicine Department have, on average, 15 specialists providing this type of care. On average, each internal medicine specialist produces about 360 outpatient visits and 120 inpatient discharges per year.

**Table 1:** Summary statistics for the variables included in the models between 1999 and 2010

Variable	General surgery				Internal medicine				Anatomical pathology			
	Mean	Std. Dev.	Min.	Max.	Mean	Std. Dev.	Min.	Max.	Mean	Std. Dev.	Min.	Max.
PHY	13	0.4	1	73	15	0.5	1	109	4	0.2	1	25
ROO	48	1.6	3	229	47	1.6	2	305	75	2.5	9	229
OUT	8 989	226.2	340	39 228	5 510	161.3	70	31 730	–	–	–	–
INP	1890	43.3	0	6 564	1862	53.7	0	11 499	–	–	–	–
SUR	1847	47	0	7 785	–	–	–	–	–	–	–	–
DIA	–	–	–	–	–	–	–	–	22 233	1 237.7	57	289 038
Number of hospitals (*i*)	112				128				55			
Number of years (*t*)	12				12				12			
Number of observations (*n*)	956				1 014				469			

Notes. PHY, number of physicians; ROO, number of rooms; OUT, number of outpatient visits; INP, number of inpatient discharges; SUR, number of performed surgeries; DIA, number of diagnostic and therapeutic procedures accomplished

For both the surgical and the medical specialties we observed that all hospitals provide outpatient consultations, but not all deliver inpatient care. This is due to the Portuguese hospital structure, where smaller hospitals are not able to provide inpatient care and patients with these needs are referred to larger facilities. It is possible to find hospitals where only 1 specialist provides healthcare services, as well as hospitals with more than 70 specialists in activity. Likewise, the discrepancy of capital levels in Portuguese hospitals is quite substantial: hospitals have an average of 50 rooms, but this number varies from 2 to more than 200.

The workload of Portuguese physicians may seem low when compared to other developed countries. In fact, in 2017, the annual number of overall consultations per physician in Portugal was only about 1000 consultations per physician, a low number when compared to the number of the average consultations per physician in the OECD countries (around 2300 consultations). The physicians’ workload for inpatient care is also below the OECD average, when measured by the number of inpatient discharges per 1000 population (110 in Portugal vs 156 in OECD countries) [34]. Although these workloads may suggest an under-utilization of the specialist workforce, it does not seem to be case of Portugal, where waiting times and waiting lists have been a concern for both health managers and decision-makers in the past two decades, and where several efforts have been made to devise and implement several recovery programs [35]. Additionally, this low workload can be justified by a number of factors, such as the low nurse ratio per population, which may suggest more physicians are required to deliver healthcare than in countries where the nurse ratio is higher. The lower number of hospital beds per 1000 population in Portugal (3.4 beds) when compared to the OECD average (4.7 beds) and the higher average length to stay (of 8.8 days in 2017, when the average for OECD countries was of 7.8 days) may also help to explain the lower number of discharges per physician in Portugal [34].

The production variable considered in the LRF for the diagnostic specialty comprises all the diagnostic procedures performed by anatomical pathologists (exams and clinical autopsies). The sample contains 469 observations from 55 different hospitals. Portuguese hospitals with an Anatomical Pathology department are the larger ones: 75 rooms, compared to an average of less than 50 for the other specialties. These hospitals employ, on average, 4 anatomical pathologists, each one producing, on average, more than 22 000 diagnostic procedures per year.

### Estimation results

The results of the LRF estimations, as well as the tests performed on the functional form and on the FE estimators are presented in Table 2. Overall, workload and capital variables included in the models are statistically relevant and the goodness-of-fit of the models is high: the LRF can explain 86%, 83%, and 61% of the variation in the number of physicians in surgical, medical and diagnostic specialties, respectively. However, individually, the estimated parameters do not have any useful interpretation and further analysis is required to obtain meaningful inferences, such as the elasticities of mean-labor use with respect to each healthcare output, the marginal rates of technical substitution of labor between the different healthcare services, and the returns to scale of labor.

**Table 2:** Estimation results for the LRF

Parameter	Variable	General surgery (*n = 956*)		Internal medicine (*n = 1014)*		Anatomical pathology (*n = 469)*	
		Coefficient	Std. Error	Coefficient	Std. Error	Coefficient	Std. Error
∝_0_	C	0.5607*	0.2475	0.1583*	0.0603	1.9218**	0.7578
∝_OUT_	ln (OUT)	− 0.4272***	0.0733	− 0.9266***	0.1203	–	–
∝_INP_	ln (INP)	− 0.1499**	0.0759	0.3064***	0.0437	–	–
∝_SUR_	ln (SUR)	0.0615	0.0683	–	–	–	–
∝_DIA_	ln (DIA)	–	–	–	–	− 0.2574***	0.1176
β_ROO_	ln (ROO)	0.2804***	0.1069	0.9156^***^	0.1418	− 1.2782**	0.1993
γ_ROO,INP_	½ ln (ROO)^*^ln (INP)	0.0113**	0.0043	0.0230	0.0165	–	–
γ_ROO,OUT_	½ ln (ROO)^*^ln (OUT)	− 0.0094	0.0125	− 0.3937***	0.0633	–	–
γ_OUT,INP_	½ ln (OUT)^*^ln (INP)	0.0076*	0.0030	− 0.1046***	0.0168	–	–
γ_ROO,SUR_	½ ln (ROO)^*^ln (SUR)	− 0.0084	0.0198	–	–	–	–
γ_SUR,INP_	½ ln (SUR)^*^ln (INP)	− 0.0190***	0.0060	–	–	–	–
γ_SUR,OUT_	½ ln (SUR)^*^ln (OUT)	0.0034	0.0126	–	–	–	–
γ_ROO,DIA_	½ ln (ROO)^*^ln (DIA)	–	–	–	–	− 0.3709***	0.0857
β_ROO,ROO_	½ [ln (ROO)]^2^	0.0240	0.0297	0.2967***	0.0407	0.8270**^*^	0.0713
∝_OUT,OUT_	½ [ln (OUT)]^2^	0.0847***	0.0083	0.2753***	0.0279	–	–
∝_INP,INP_	½ [ln (INP)]^2^	0.0488***	0.0110	0.0629***	0.0062	–	–
∝_SUR,SUR_	½ [ln (SUR)]^2^	0.0331***	0.0117	–	–	–	–
∝_DIA,DIA_	½ [ln (DIA)]^2^	–	–	–	–	0.1537***	0.0175
*R* ^2^		0.8621		0.8326		0.6100	
Adjusted *R*^2^		0.8584		0.8293		0.6057	
*F-statistic*		232.6221***		247.0297***		144.7795***	
Redundant fixed effects test							
*F-statistic*		5.7358***		1.8425**		1.6236*	
Redundant (2nd order) variables test							
*F-statistic*		38.0364***		39.9792***		44.6099***	

*p < 0.10, **p < 0.05, ***p < 0.01

The test on the joint significance of the second-order terms shows that these terms are statistically significant and relevant to the model, which we confirm by rejecting the null hypothesis of redundancy of the second-order terms with a significance level of 5%. This means that the flexible functional form considered is superior to the traditional Cobb-Douglas form, for all specialties.

Additionally, we confirm that considering year FE is a valid approach for the medical and the surgical specialties: at the 5% significance level, the test on the FE redundancy shows that FE are statistically different from zero, thus affecting the production of healthcare services in these specialties. As no statistically significant FE were found (at the 5% significance level) for Anatomical Pathology, we did not go further with the productivity analysis for this specialty.

All the estimations and tests were performed using the statistical software EViews®8.

### Labor productivity

**Fig. 1:**
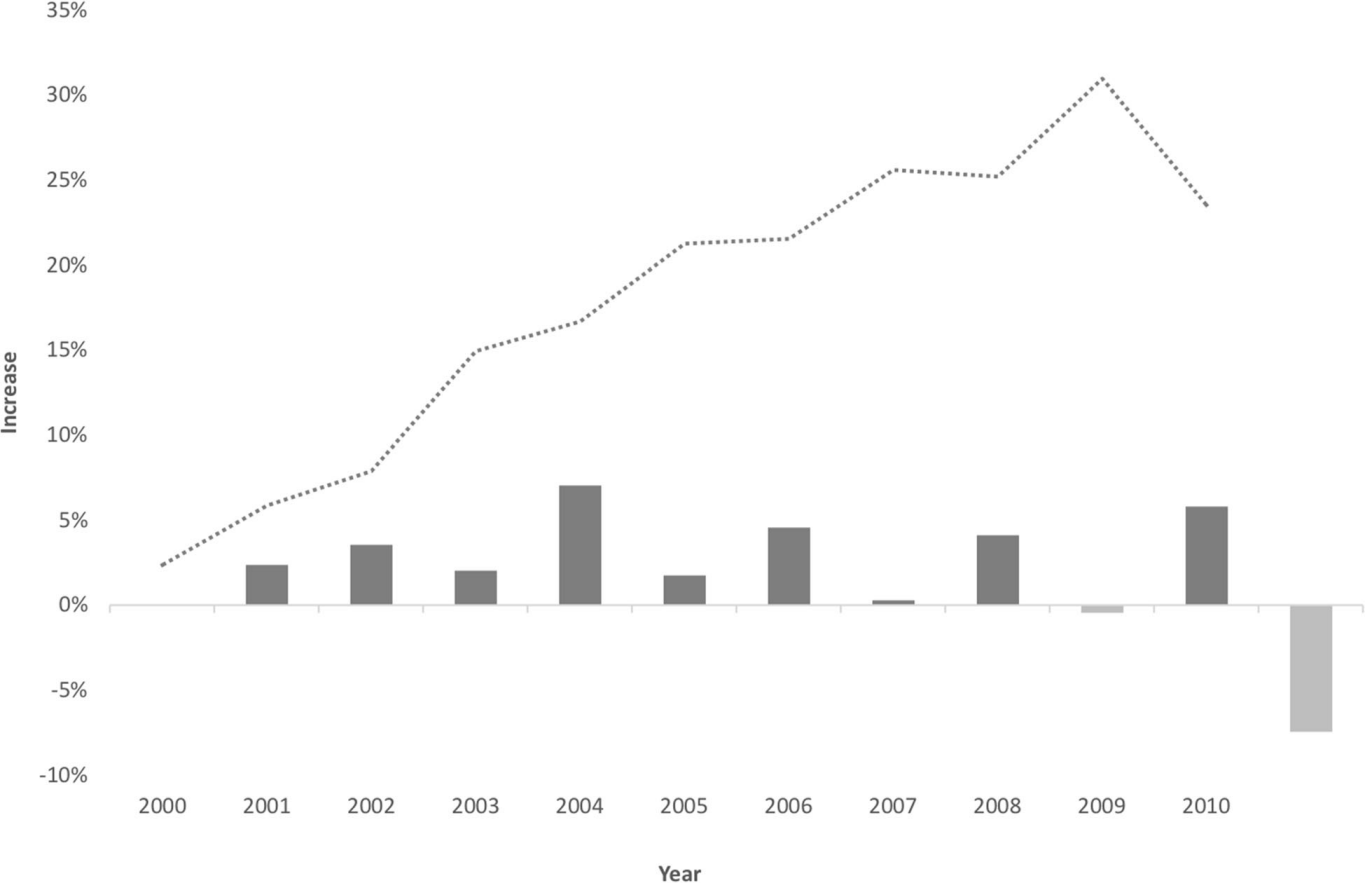
Technical change rate and cumulative technical progress in general surgery over 11 years

**Fig. 2:**
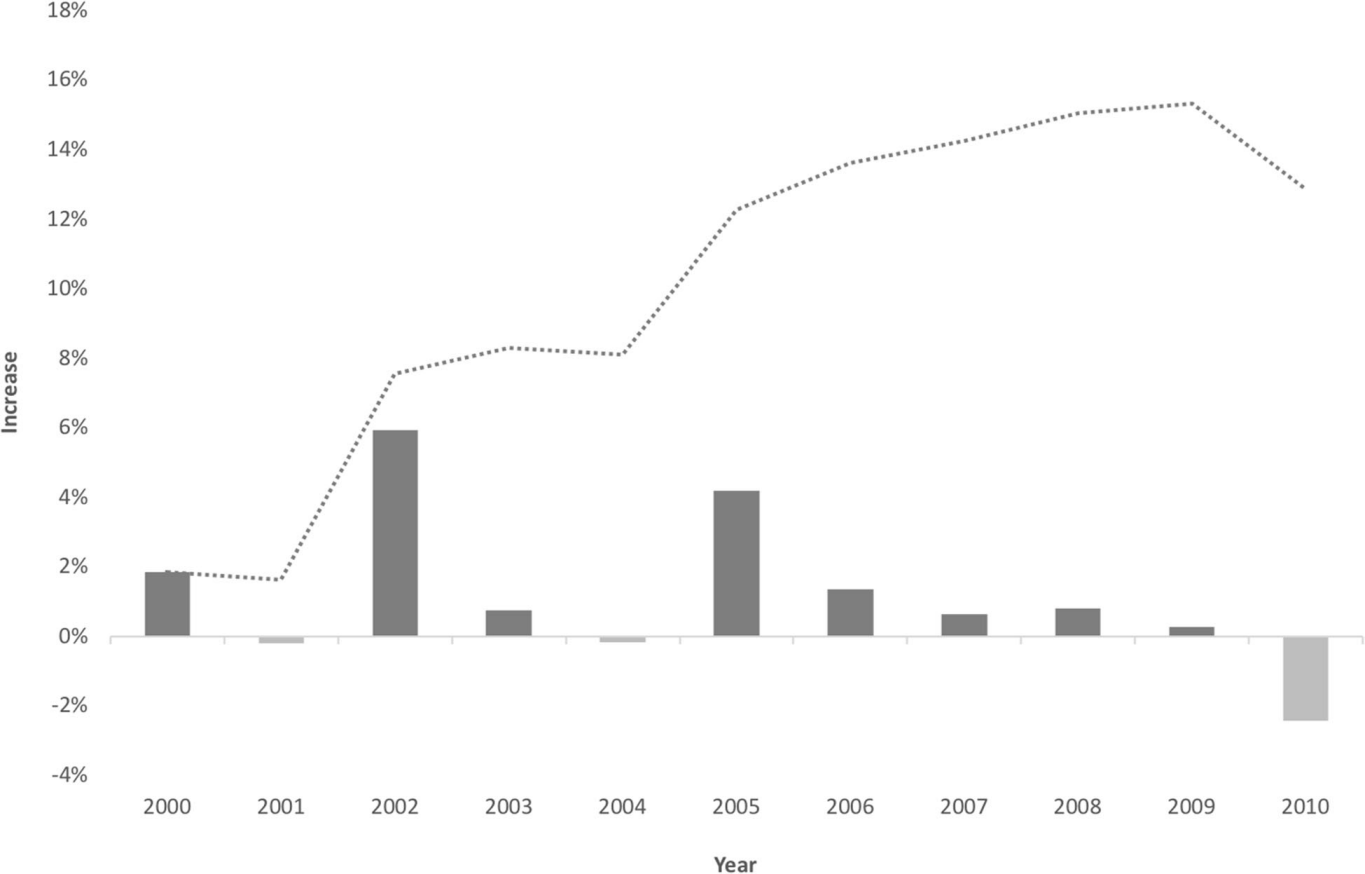
Technical change rate and cumulative technical progress in internal medicine over 11 years

To analyze the technical progress and the labor productivity we used the FE estimates (Additional file 1: Table S3). FE decreases can be interpreted as technical progress (decreasing labor demand) and increases as technical regress (increasing demand for labor). Results on both the annual technical change rate and the cumulative technological progress in healthcare delivery are presented in Fig. 1 (general surgery) and Fig. 2 (internal medicine).

There were only two periods of regression for the surgical specialty (2007–2008 and 2009–2010) and three for the medical specialty (2000–2001, 2003–2004, and 2009–2010), where a decreasing labor productivity was apparent. The 2009–2010 period, however, had a sharper reduction in productivity. This decrease may be related to the evolution of the mean number of physicians and the possible existence of economies of scale, as it is further explained in the discussion section. Increasing RTS would help to explain this phenomenon, as no other structural or political change occurred that could explain significant changes in productivity.

Our results show also that healthcare delivery has benefited from an increasing productivity of physicians’ labor of around 2.14% per year for General Surgery. In the case of Internal Medicine the improvement was smaller, of around 1.20%.

### Elasticities of labor use, marginal rates of technical substitution, and returns to scale

The estimation results were used to further analyze the healthcare services delivered by physicians. The elasticities of labor use for each production line, the marginal rates of technical substitution (MRTS) between healthcare services and the labor RTS are presented in Table 3.

**Table 3:** Elasticities of mean-labor use, MRTS and RTS

Year	General surgery							Internal medicine				Anatomical pathology	
	*E*_out_	*E*_inp_	*E*_sur_	MRTS_out, inp_	MRTS_out, sur_	MRTS_inp, sur_	RTS	*E*_out_	*E*_inp_	MRTS_out, inp_	RTS	*E*_*dia*_	RTS
1999	0.3353	0.1668	0.1528	2.0099	2.1942	1.0917	1.5267	0.2788	0.3529	0.7900	1.5831	0.6271	1.5946
2000	0.3359	0.1629	0.1544	2.0620	2.1756	1.0551	1.5309	0.2857	0.3454	0.8270	1.5845	0.6040	1.6557
2001	0.3397	0.1639	0.1600	2.0731	2.1235	1.0243	1.5071	0.3042	0.3447	0.8827	1.5411	0.6419	1.5578
2002	0.3486	0.1633	0.1600	2.1354	2.1794	1.0206	1.4884	0.3116	0.3253	0.9577	1.5700	0.6475	1.5443
2003	0.3551	0.1615	0.1618	2.1994	2.1943	0.9977	1.4741	0.3161	0.3283	0.9630	1.5518	0.6550	1.5266
2004	0.3570	0.1612	0.1584	2.2153	2.2546	1.0177	1.4781	0.3229	0.3173	1.0176	1.5622	0.6590	1.5174
2005	0.3601	0.1625	0.1607	2.2164	2.2410	1.0111	1.4634	0.3107	0.3156	0.9845	1.5967	0.6702	1.4921
2006	0.3701	0.1682	0.1629	2.2006	2.2718	1.0324	1.4263	0.3277	0.3126	1.0484	1.5619	0.6182	1.6175
2007	0.3566	0.1672	0.1606	2.1330	2.2205	1.0410	1.4613	0.2963	0.3045	0.9730	1.6645	0.5971	1.6748
2008	0.3756	0.1625	0.1645	2.3118	2.2831	0.9876	1.4233	0.3176	0.3097	1.0254	1.5941	0.5930	1.6862
2009	0.3724	0.1609	0.1704	2.3142	2.1857	0.9445	1.4209	0.3329	0.3003	1.1088	1.5793	0.6162	1.6280
2010	0.3480	0.1529	0.1642	2.2763	2.1194	0.9311	1.5036	0.3402	0.2933	1.1600	1.5784	0.6340	1.5774
1999–2010	0.3536	0.1628	0.1605	2.1713	2.2025	1.0144	1.4772	0.3113	0.3220	0.9670	1.5791	0.6313	1.5841

Results show that, for all specialties under analysis, elasticities of mean labor-use are relatively stable over the years, although it was verified a slight increase of surgeries and outpatient visits and a decrease of inpatient discharges’ weight over time.

The general surgery elasticities for the overall period show that, ceteris paribus, increasing outpatient visits by 1% requires 0.35% more physicians; increasing the number of inpatient discharges by 1% requires 0.16% additional specialists delivering care; and increasing the surgeries by 1% requires 0.16% more general surgeons. These results mean that one additional physician is needed to increase the outpatient care by 1975 visits, the inpatient care by 909 discharges or the surgeries by 888 surgeries.

From the MRTS, which can be interpreted as the opportunity costs of the physicians’ time, we found that increasing the outpatient visits delivered by 1% (90 visits) would imply a reduction of 2.17% in inpatient discharges (41 discharges) or, alternatively, the sacrifice of 2.20% of surgeries (4 surgeries).

The internal medicine results suggest that, ceteris paribus, an increase in the outpatient visits by 1% requires 0.31% more specialists delivering care and an increase in the number of inpatient discharges by 1% requires 0.32% additional physicians. These results mean that one additional physician would be able to increase the outpatient care by 1 184 visits or the inpatient care by 388 discharges. The MRTS between outpatient visits and inpatient discharges for this specialty shows that increasing the outpatient visits by 1% (55 visits) would reduce inpatient care in 0.97% (18 discharges) as a trade-off.

For the diagnostic specialty we found that, ceteris paribus, increasing the number of diagnose procedures by 1% requires 0.63% additional physicians: one additional physician would increase the number of procedures performed by 8 823.

Additionally, results show RTS for the overall period of 1.48 for the surgical specialty, and of 1.58 for both the medical and the diagnostic specialties, meaning that the production of healthcare services has increasing RTS. As a result, increasing all healthcare services produced by 1% would lead to an additional requirement of physicians less than proportional: 0.68% for the surgical specialty and 0.63% for the other specialties.

## Discussion

Ensuring an efficient delivery of healthcare services is crucially dependent on both the prediction of the future demand for healthcare services and on planning the HHR needed to properly deliver these services.

Considering this, we proposed an LRF relating the number of physicians with a set of specialty-specific workload and capital variables to empirically quantify and describe the relation between healthcare services and the HHR needed. The method is based on the assumption that health managers and decision-makers can size and adjust the level of the HHR in response to a given expected demand, which is a very realistic assumption.

We considered a flexible form, which we also found to be superior to the traditional Cobb-Douglas form, confirming the results obtained by other authors [36]. Additionally, we use period FE estimation to account for the technological progress, concluding that there are statistically significant time effects affecting the production of healthcare services, which allows for the analysis of labor productivity and its impact on the healthcare services delivered over time.

### Key findings

Most of the health workforce planning models include productivity as a factor influencing the future workforce needs, usually by making some arbitrary assumptions regarding productivity growth rates [2]. Our models estimate these rates, and overall results show that the delivery of healthcare has benefited from an increasing productivity of physicians’ labor. These results are in line with other international studies on healthcare productivity, suggesting that the positive impact of technological innovations on labor productivity exceeds the negative effect that may derive from the increased patient complexity experienced in the healthcare sector [36, 37]. For the medical specialty the improvement was smaller, which can be explained by the larger dependency of surgical specialties on technology [38] and by a program recently implemented in Portugal to reduce the waiting times for surgeries, which is based on additional financial incentives to hospitals that perform the surgeries on time, and penalties for those which do not.

The production of healthcare services in Portugal has increasing returns to scale: increasing the health workforce tends to originate a more than proportional increase in the level of healthcare services delivered, which is in line with previous results for Portugal [39] and with several other studies that showed increasing returns to scale among median-size hospitals [37]. These results suggest that the Portuguese specialists would be more productive on delivering healthcare services if hospitals had an higher HHR concentration, which is interesting regarding that the largest hospitals are usually the ones dealing with the higher diversity, severity and complexity of cases. Additionally, the interaction between returns to scale and the evolution of the mean number of physicians may also explain changes in productivity: in case of increasing returns to scale, increasing the amount of labor increases the labor productivity and decreasing the amount of labor leads to a productivity decrease, while in case of decreasing returns to scale, the changes in the labor levels have exactly the opposite effect in productivity. Thus, these results explain the decreasing productivity registered between 2009 and 2010, where the average number of specialists decreased due to retirements, followed by a significant decrease in productivity.

The elasticities of mean labor use, which reflect the portion of time that a physician spends on each type of activity, are relatively stable over the years. Notwithstanding, it is possible to verify a slight increase of surgeries and outpatient visits and a decrease of inpatient discharges’ weight over time. These changes follow the efforts that have been made in Portugal for substituting inpatient care with outpatient care, with the aim of controlling both health expenditures and avoidable hospital infections [40]. Additionally, they are also a consequence of the recent increase in the number of surgical procedures carried out on a same-day basis (ambulatory surgery), similar to what happened in other countries [34].

### Limitations and further work

The application of this method to other datasets would be interesting for comparing resources allocation patterns across countries, regions or health systems. Further applications of the proposed method in different contexts (e.g., where some of the explanatory variables are not so highly correlated as in the Portuguese case), or assuming the number of nurses as the dependent variable of the models (e.g., for cases where the health system is characterized by higher nursing ratios and lower physician ratios per population) would also be interesting.

Notwithstanding, future empirical studies applying the proposed method should have present the main limitations of our empirical data: full-time equivalents (FTE) should be used instead of the number of HHR and the volume of healthcare services provided can be adjusted to account for diversity and complexity, using relative measures such as the Case Mix Index (CMI).

Moreover, further work on HHR productivity can be undertaken to provide a better understanding on the main factors driving the changes in productivity that were found.

### Implications for policy and practice

The proposed method yields simple yet powerful models to understand and quantify the relation between healthcare services and HHR needed. Estimation of HHR requirements based on our models can be performed as the second step—that aims to translate services to HHR—on the different demand approaches to HHR planning. These models can be applied both for a single medical specialty and for a group of specialties, at international, national or regional level.

Additionally, by empirically quantifying the relation between HHR and healthcare services produced, this method can be used to complement several other existent approaches aiming to translate healthcare services in HHR, which require the use of service standards—measured in unit time or rate of work—as an input, to further estimate the HHR requirements (e.g., WHO Workload Indicators of Staffing Needs—WISN) [41]. These service standards, which may be derived from an empirical method like the one proposed in this paper, may also be used: (i) to conduct international or regional comparisons of HHR productivity; (ii) as a benchmark for specific hospitals; or (iii) to measure and monitor the distance to some ideal/desirable productivity pattern.

Our approach provides several interesting insights that can be very useful to health managers and policy-makers, such as:

- The variation in the number of physicians that is required to meet fluctuations in the demand for healthcare, to ensure that enough resources will exist to provide healthcare services to the patients who need them;
- The proportion of time spent by physicians in each type of care provided, both to elucidate about the distribution of HHR workload, and to assist in the implementation and monitoring of specific strategies related to healthcare delivery (e.g., reduce the inpatient care, increase ambulatory surgeries);
- The feasible allocations of the available physicians between different healthcare services, based on the opportunity cost of physicians’ labor, informing on the allocations that most benefits the population and the organizations;
- The effect of the HHR concentration in hospitals. Based on the returns to scale of labor provided, this method informs on the effect of changes in the scale of hospitals’ labor use and points the HHR concentration changes that may lead to a higher productivity of human resources on delivering healthcare services;
- The evolution of the labor productivity in hospitals. The time fixed-effects estimates are useful to predict the adjustments in the human resources levels that may be required over the time, due to the evolution of labor productivity.

Overall, this method can be used to enable a more informed sizing and allocation of human resources and to achieve a better HHR management in hospitals, which is highly dependent on reliable HHR estimations; to inform on the right scale of health facilities and HHR concentration; and to foster a deeper knowledge on the evolution of the labor productivity and on the opportunity costs of labor in different healthcare services.

## Additional file

Additional file 1: Models, formulas and intermediary results. (DOCX 86 kb)

## Abbreviations

CMI: Case Mix Index
FE: Fixed effects
FTE: Full-time equivalent
HHR: Health Human Resources
INE: Portuguese Institute of Statistics
IRF: Input requirements function
LRF: Labor requirements function
MRTS: Marginal rates of technical substitution
PF: Production function
RTS: Returns to scale
