# Supplementary material for: A labor requirements function for sizing the health workforce
Source: Hum Resour Health. 2018 Dec 4;16:67. doi: 10.1186/s12960-018-0334-4 (PMC6278005; doi:10.1186/s12960-018-0334-4)
Supplement: Supplementary file 1 — Models, formulas and intermediary results. (DOCX 86 kb) [file 12960_2018_334_MOESM1_ESM.docx]

**Additional file 1**

***Model for surgical specialties*:**

(A.1)

${ln(PHY {}_{i,t})=}\propto_{0}+ \propto_{1} {ln(OUT}_{i,t})+ \propto_{2} {ln(INP}_{i,t})+ \propto_{3} {ln(SUR}_{i,t})+\beta_{1} {ln(ROO}_{i,t})+\frac{1}{2} \gamma_{1,1} \ln{(ROO}_{i,t})*{ln(OUT}_{i,t}) +\frac{1}{2} \gamma_{1,2} {\ln(ROO}_{i,t})*{ln(INP}_{i,t}) + \frac{1}{2} \gamma_{1,3} \ln{(ROO}_{i,t})*{ln(SUR}_{i,t})+\frac{1}{2} \propto_{1,2} {\ln(OUT}_{i,t})*ln({INP}_{i,t})+ \frac{1}{2} \propto_{1,3} \ln{(OUT}_{i,t})*{ln(SUR}_{i,t}) +\frac{1}{2} \propto_{2,3} {\ln(INP}_{i,t})*{ln(SUR}_{i,t}) + \frac{1}{2} \propto_{1,1} [ln({{OUT}}_{i,t})]^{2} +\frac{1}{2} \propto_{2,2} {{[ln(INP}}_{i,t})]^{2} +\frac{1}{2} \propto_{3,3} {{[ln(SUR}}_{i,t})]^{2}+ \frac{1}{2} \beta_{1,1} {{[ln(ROO}}_{i,t})]^{2}+ \delta_{t}+ \mu_{i,t}$

***Model for medical specialties*:**

(A.2)

${ln(PHY {}_{i,t})=}\propto_{0}+ \propto_{1} {ln(OUT}_{i,t})+ \propto_{2} {ln(INP}_{i,t})+\beta_{1} {ln(ROO}_{i,t})+\frac{1}{2} \gamma_{1,1} {ln(ROO}_{i,t})*{ln(OUT}_{i,t}) +\frac{1}{2} \gamma_{1,2} {ln(ROO}_{i,t})*{ln(INP}_{i,t}) + \frac{1}{2} \propto_{1,2} {ln(OUT}_{i,t})* {\ln(INP}_{i,t})+\frac{1}{2} \propto_{1,1} [\ln({{OUT}}_{i,t})]^{2} +\frac{1}{2} \propto_{2,2} {{[\ln(INP}}_{i,t})]^{2} + \frac{1}{2} \beta_{1,1} {{[\ln(ROO}}_{i,t})]^{2}+\delta_{t}+\mu_{i,t}$

***Model for diagnostic specialties*:**

(A.3)

${ln(PHY {}_{i,t})=}\propto_{0}+ \propto_{1} {ln(DIA}_{i,t})+ \beta_{1} {ln(ROO}_{i,t})+\frac{1}{2} \gamma_{1,1} ln{(ROO}_{i,t})*{ln(DIA}_{i,t}) +\frac{1}{2} \propto_{1,1} {{[ln(DIA}}_{i,t})]^{2}+ \frac{1}{2} \beta_{1,1} {{[ln(ROO}}_{i,t})]^{2}+{\delta_{t}+\mu}_{i,t}$,

where PHY is the number of physicians, ROO is the number of rooms (consultation rooms, operating rooms and yards), OUT is the number of outpatient visits, INP is the number of inpatient discharges, SUR is the number of surgeries, DIA is the number of diagnostic and therapeutic procedures, δ stands for a set of binary year variables (each multiplied by their respective regression coefficients ), $\mu$ represents the error term of the model, $i$the index for the hospital and $t$the index for the period.

***Elasticity of mean labor used in the production of output*** $\boldsymbol{Y}_{\boldsymbol{i}}$**:**

(A.4)

$E_{i}=\frac{\partial\ln L}{\partial\ln Y_{i}}= \propto_{i} +\frac{1}{2}\sum_{j=1}^{n} \propto_{i,j}\ln\left( Y_{j} \right)+ \frac{1}{2}\sum_{k=1}^{m} \gamma_{ik}\ln\left( K_{k} \right)$.

***Marginal Rate of Technical Substitution between two HS, i and j*:**

(A.5)

${MRTS}_{ij}=\frac{E_{i}}{E_{j}}$.

***Returns to scale (RTS) of labor:***

(A.6)

${RTS}=\left( \sum_{i=1}^{n} E_{i} \right)^{-1}$.

***Original model for surgical specialties*:**

(A.7)

${ln(PHY {}_{i,t})=}\propto_{0}+ \propto_{1} {ln(OUT}_{i,t})+ \propto_{2} {ln(INP}_{i,t})+ \propto_{3} {ln(SUR}_{i,t})+\beta_{1} {ln(ROO}_{i,t})+ \beta_{2} {ln(NUR}_{i,t})+\beta_{3} {ln(RES}_{i,t})+\beta_{4} {ln(OTH}_{i,t})+\beta_{5} {ln(BED}_{i,t})+ \frac{1}{2} \gamma_{1,2} {\ln(ROO}_{i,t})*{ln(INP}_{i,t}) +\frac{1}{2} \gamma_{2,2} {\ln(NUR}_{i,t})*{ln(INP}_{i,t})+ \frac{1}{2} \gamma_{3,2} {\ln(RES}_{i,t})*{ln(INP}_{i,t}) +\frac{1}{2} \gamma_{4,2} {\ln(OTH}_{i,t})*{ln(INP}_{i,t}) +\frac{1}{2} \gamma_{5,2} {\ln(BED}_{i,t})*{ln(INP}_{i,t})+ \frac{1}{2} \gamma_{1,1} {\ln(ROO}_{i,t})*{ln(OUT}_{i,t}) +\frac{1}{2} \gamma_{2,1} {\ln(NUR}_{i,t})*{ln(OUT}_{i,t}) +\frac{1}{2} \gamma_{3,1} {\ln(RES}_{i,t})*{ln(OUT}_{i,t}) +\frac{1}{2} \gamma_{4,1} {\ln(OTH}_{i,t})*{ln(OUT}_{i,t}) +\frac{1}{2} \gamma_{5,1} {\ln(BED}_{i,t})*{ln(OUT}_{i,t}) +\frac{1}{2} \gamma_{1,3} {\ln(ROO}_{i,t})*{ln(SUR}_{i,t}) +\frac{1}{2} \gamma_{2,3} {\ln(NUR}_{i,t})*{ln(SUR}_{i,t})+ \frac{1}{2} \gamma_{3,3} {\ln(RES}_{i,t})*{ln(SUR}_{i,t}) +\frac{1}{2} \gamma_{4,3} {\ln(OTH}_{i,t})*{ln(SUR}_{i,t}) +\frac{1}{2} \gamma_{5,3} {\ln(BED}_{i,t})*{ln(SUR}_{i,t}) + \frac{1}{2} \propto_{1,3} \ln{(OUT}_{i,t})*{ln(SUR}_{i,t}) +\frac{1}{2} \propto_{1,2} {\ln(OUT}_{i,t})*ln({INP}_{i,t})+ \frac{1}{2} \propto_{2,3} {\ln(INP}_{i,t})*{ln(SUR}_{i,t})+ \frac{1}{2} \beta_{1,2} {\ln(ROO}_{i,t})*{ln(NUR}_{i,t}) +\frac{1}{2} \beta_{1,3} {\ln(ROO}_{i,t})*{ln(RES}_{i,t}) +\frac{1}{2} \beta_{1,4} {\ln(ROO}_{i,t})*{ln(OTH}_{i,t}) +\frac{1}{2} \beta_{1,5} {\ln(ROO}_{i,t})*{ln(BED}_{i,t}) +\frac{1}{2} \beta_{2,3} {\ln(NUR}_{i,t})*{ln(RES}_{i,t}) +\frac{1}{2} \beta_{2,4} {\ln(NUR}_{i,t})*{ln(OTH}_{i,t}) +\frac{1}{2} \beta_{2,5} {\ln(NUR}_{i,t})*{ln(BED}_{i,t}) +\frac{1}{2} \beta_{3,4} {\ln(RES}_{i,t})*{ln(OTH}_{i,t}) +\frac{1}{2} \beta_{3,5} {\ln(RES}_{i,t})*{ln(BED}_{i,t}) +\frac{1}{2} \beta_{4,5} {\ln(OTH}_{i,t})*{ln(BED}_{i,t}) + \frac{1}{2} \propto_{1,1} [ln({{OUT}}_{i,t})]^{2} +\frac{1}{2} \propto_{2,2} {{[ln(INP}}_{i,t})]^{2} +\frac{1}{2} \propto_{3,3} {{[ln(SUR}}_{i,t})]^{2}+ \frac{1}{2} {{\beta_{1,1}[ln(ROO}}_{i,t})]^{2}+ \frac{1}{2} {{\beta_{2,2}[ln(NUR}}_{i,t})]^{2}+ \frac{1}{2} {{\beta_{3,3}[ln(RES}}_{i,t})]^{2}+ \frac{1}{2} {{\beta_{4,4}[ln(OTH}}_{i,t})]^{2}+ \frac{1}{2} {{\beta_{5,5}[ln(BED}}_{i,t})]^{2}+ \delta_{t}+ \mu_{i,t}$

***Original model for medical specialties*:**

(A.8)

${ln(PHY {}_{i,t})=}\propto_{0}+ \propto_{1} {ln(OUT}_{i,t})+ \propto_{2} {ln(INP}_{i,t})+\beta_{1} {ln(ROO}_{i,t})+ \beta_{2} {ln(NUR}_{i,t})+\beta_{3} {ln(RES}_{i,t})+\beta_{4} {ln(OTH}_{i,t})+\beta_{5} {ln(BED}_{i,t})+ \frac{1}{2} \gamma_{1,2} {\ln(ROO}_{i,t})*{ln(INP}_{i,t}) + \frac{1}{2} \gamma_{2,2} {\ln(NUR}_{i,t})*{ln(INP}_{i,t})+ \frac{1}{2} \gamma_{3,2} {\ln(RES}_{i,t})*{ln(INP}_{i,t}) +\frac{1}{2} \gamma_{4,2} {\ln(OTH}_{i,t})*{ln(INP}_{i,t}) +\frac{1}{2} \gamma_{5,2} {\ln(BED}_{i,t})*{ln(INP}_{i,t})+ \frac{1}{2} \gamma_{1,1} {\ln(ROO}_{i,t})*{ln(OUT}_{i,t}) +\frac{1}{2} \gamma_{2,1} {\ln(NUR}_{i,t})*{ln(OUT}_{i,t}) +\frac{1}{2} \gamma_{3,1} {\ln(RES}_{i,t})*{ln(OUT}_{i,t}) +\frac{1}{2} \gamma_{4,1} {\ln(OTH}_{i,t})*{ln(OUT}_{i,t}) +\frac{1}{2} \gamma_{5,1} {\ln(BED}_{i,t})*{ln(OUT}_{i,t}) +\frac{1}{2} \propto_{1,2} {\ln(OUT}_{i,t})*ln({INP}_{i,t})+ \frac{1}{2} \beta_{1,2} {\ln(ROO}_{i,t})*{ln(NUR}_{i,t}) +\frac{1}{2} \beta_{1,3} {\ln(ROO}_{i,t})*{ln(RES}_{i,t}) +\frac{1}{2} \beta_{1,4} {\ln(ROO}_{i,t})*{ln(OTH}_{i,t}) +\frac{1}{2} \beta_{1,5} {\ln(ROO}_{i,t})*{ln(BED}_{i,t}) +\frac{1}{2} \beta_{2,3} {\ln(NUR}_{i,t})*{ln(RES}_{i,t}) +\frac{1}{2} \beta_{2,4} {\ln(NUR}_{i,t})*{ln(OTH}_{i,t}) +\frac{1}{2} \beta_{2,5} {\ln(NUR}_{i,t})*{ln(BED}_{i,t}) +\frac{1}{2} \beta_{3,4} {\ln(RES}_{i,t})*{ln(OTH}_{i,t}) +\frac{1}{2} \beta_{3,5} {\ln(RES}_{i,t})*{ln(BED}_{i,t}) +\frac{1}{2} \beta_{4,5} {\ln(OTH}_{i,t})*{ln(BED}_{i,t})+ \frac{1}{2} \propto_{1,1} [ln({{OUT}}_{i,t})]^{2} +\frac{1}{2} \propto_{2,2} {{[ln(INP}}_{i,t})]^{2}+ \frac{1}{2} {{\beta_{1,1}[ln(ROO}}_{i,t})]^{2}+ \frac{1}{2} {{\beta_{2,2}[ln(NUR}}_{i,t})]^{2}+ \frac{1}{2} {{\beta_{3,3}[ln(RES}}_{i,t})]^{2}+ \frac{1}{2} {{\beta_{4,4}[ln(OTH}}_{i,t})]^{2}+ \frac{1}{2} {{\beta_{5,5}[ln(BED}}_{i,t})]^{2}+ \delta_{t}+ \mu_{i,t}$

***Original model for diagnostic specialties*:**

(A.9)

${ln(PHY {}_{i,t})=}\propto_{0}+ \propto_{1} {ln(DIA}_{i,t})+\beta_{1} {ln(ROO}_{i,t})+ \beta_{2} {ln(NUR}_{i,t})+\beta_{3} {ln(RES}_{i,t})+\beta_{4} {ln(OTH}_{i,t})+\beta_{5} {ln(BED}_{i,t})+ \frac{1}{2} \gamma_{1,1} {\ln(ROO}_{i,t})*{ln(DIA}_{i,t}) +\frac{1}{2} \gamma_{2,1} {\ln(NUR}_{i,t})*{ln(DIA}_{i,t}) +\frac{1}{2} \gamma_{3,1} {\ln(RES}_{i,t})*{ln(DIA}_{i,t}) +\frac{1}{2} \gamma_{4,1} {\ln(OTH}_{i,t})*{ln(DIA}_{i,t}) +\frac{1}{2} \gamma_{5,1} {\ln(BED}_{i,t})*{ln(DIA}_{i,t}) +\frac{1}{2} \beta_{1,2} {\ln(ROO}_{i,t})*{ln(NUR}_{i,t}) +\frac{1}{2} \beta_{1,3} {\ln(ROO}_{i,t})*{ln(RES}_{i,t}) +\frac{1}{2} \beta_{1,4} {\ln(ROO}_{i,t})*{ln(OTH}_{i,t}) +\frac{1}{2} \beta_{1,5} {\ln(ROO}_{i,t})*{ln(BED}_{i,t}) +\frac{1}{2} \beta_{2,3} {\ln(NUR}_{i,t})*{ln(RES}_{i,t}) +\frac{1}{2} \beta_{2,4} {\ln(NUR}_{i,t})*{ln(OTH}_{i,t}) +\frac{1}{2} \beta_{2,5} {\ln(NUR}_{i,t})*{ln(BED}_{i,t}) +\frac{1}{2} \beta_{3,4} {\ln(RES}_{i,t})*{ln(OTH}_{i,t}) +\frac{1}{2} \beta_{3,5} {\ln(RES}_{i,t})*{ln(BED}_{i,t}) +\frac{1}{2} \beta_{4,5} {\ln(OTH}_{i,t})*{ln(BED}_{i,t})+ \frac{1}{2} \propto_{1,1} [ln({{DIA}}_{i,t})]^{2} + \frac{1}{2} {{\beta_{1,1}[ln(ROO}}_{i,t})]^{2}+ \frac{1}{2} {{\beta_{2,2}[ln(NUR}}_{i,t})]^{2}+ \frac{1}{2} {{\beta_{3,3}[ln(RES}}_{i,t})]^{2}+ \frac{1}{2} {{\beta_{4,4}[ln(OTH}}_{i,t})]^{2}+ \frac{1}{2} {{\beta_{5,5}[ln(BED}}_{i,t})]^{2}+ \delta_{t}+ \mu_{i,t}$

where PHY is the number of physicians, ROO is the number of rooms (consultation rooms, operating rooms and yards), OUT is the number of outpatient visits, INP is the number of inpatient discharges, SUR is the number of surgeries, DIA is the number of diagnostic and therapeutic procedures, NUR is the number of nurses, RES is the number of residents, OTH is the number of other health professionals, BED is the number of inpatient beds, δ stands for a set of binary year variables (each multiplied by their respective regression coefficients ), $\mu$ represents the error term of the model, $i$the index for the hospital and $t$the index for the period.

**Table S1**- Mean-values of the explanatory variables

| Year |  | General Surgery | | | |  | Internal Medicine | | |  | Anatomical Pathology | |
| --- | --- | --- | --- | --- | --- | --- | --- | --- | --- | --- | --- | --- |
|  |  | **ln (OUT)** | **ln (INP)** | **ln (ROO)** | **ln (SUR)** |  | **ln (OUT)** | **ln (INP)** | **ln (ROO)** |  | **ln (ROO)** | **ln (DIA)** |
| 1999 |  | 8,4345 | 7,1038 | 3,0430 | 6,7608 |  | 7,8646 | 6,7161 | 3,0897 |  | 3,1354 | 9,5373 |
| 2000 |  | 8,4563 | 7,0084 | 3,1013 | 6,7665 |  | 7,9180 | 6,6306 | 3,1521 |  | 3,2272 | 9,4974 |
| 2001 |  | 8,4919 | 7,0963 | 3,1747 | 7,0002 |  | 8,0159 | 6,6953 | 3,1775 |  | 3,2619 | 9,7862 |
| 2002 |  | 8,6056 | 7,0482 | 3,1973 | 6,9668 |  | 8,0100 | 6,376 | 3,2167 |  | 3,2735 | 9,8368 |
| 2003 |  | 8,6948 | 6,9923 | 3,2791 | 7,0030 |  | 8,1350 | 6,5051 | 3,3342 |  | 3,3491 | 9,9767 |
| 2004 |  | 8,7340 | 6,9157 | 3,3155 | 6,8591 |  | 8,1316 | 6,3258 | 3,3429 |  | 3,3512 | 10,0052 |
| 2005 |  | 8,7682 | 6,9681 | 3,3735 | 6,9719 |  | 8,1292 | 6,2844 | 3,4122 |  | 3,4139 | 10,1535 |
| 2006 |  | 8,8815 | 7,0998 | 3,5047 | 7,1353 |  | 8,3178 | 6,3643 | 3,5685 |  | 3,4299 | 9,8348 |
| 2007 |  | 8,7428 | 7,0522 | 3,6310 | 7,0845 |  | 8,1896 | 6,1215 | 3,6134 |  | 3,5786 | 9,8766 |
| 2008 |  | 8,9849 | 6,9167 | 3,6898 | 7,1161 |  | 8,3374 | 6,3192 | 3,6592 |  | 3,7287 | 10,0313 |
| 2009 |  | 8,9459 | 6,9581 | 3,7896 | 7,3472 |  | 8,4628 | 6,2519 | 3,7745 |  | 3,7523 | 10,1977 |
| 2010 |  | 8,6552 | 6,7738 | 3,5017 | 7,0100 |  | 8,1541 | 5,9558 | 3,3844 |  | 3,5639 | 10,0988 |
| 1999-2010 |  | **8,6859** | **6,9973** | **3,3612** | **6,9885** |  | **8,1252** | **6,3890** | **3,3757** |  | **3,4074** | **9,8925** |

**Table S2**- Correlation Matrix

|  | **BEDS** | **BEDS** GS | **BEDS** IM | **ROO** | **PHY**  AP | **PHY**  GS | **PHY**  IM | **RES** | **NUR** | **OTH** | **INP**  GS | **INP**  IM | **OUT**  GS | **OUT**  IM | **SUR**  GS | **DIA**  AP |
| --- | --- | --- | --- | --- | --- | --- | --- | --- | --- | --- | --- | --- | --- | --- | --- | --- |
| **BEDS** | 1,000 |  |  |  |  |  |  |  |  |  |  |  |  |  |  |  |
| **BEDS** GS | 0,927 | 1,000 |  |  |  |  |  |  |  |  |  |  |  |  |  |  |
| **BEDS** IM | 0,849 | 0,885 | 1,000 |  |  |  |  |  |  |  |  |  |  |  |  |  |
| **ROO** | **0,902** | 0,784 | 0,705 | 1,000 |  |  |  |  |  |  |  |  |  |  |  |  |
| **PHY** AP | 0,697 | 0,652 | 0,649 | 0,793 | 1,000 |  |  |  |  |  |  |  |  |  |  |  |
| **PHY** GS | 0,799 | 0,843 | 0,824 | 0,790 | 0,754 | 1,000 |  |  |  |  |  |  |  |  |  |  |
| **PHY** IM | 0,772 | 0,785 | 0,856 | 0,751 | 0,693 | 0,786 | 1,000 |  |  |  |  |  |  |  |  |  |
| **RES** | 0,826 | 0,764 | 0,712 | **0,941** | 0,783 | 0,785 | 0,773 | 1,000 |  |  |  |  |  |  |  |  |
| **NUR** | 0,882 | 0,826 | 0,766 | **0,921** | 0,715 | 0,804 | 0,769 | 0,831 | 1,000 |  |  |  |  |  |  |  |
| **OTH** | 0,870 | 0,813 | 0,771 | **0,927** | 0,772 | 0,835 | 0,806 | 0,844 | 0,890 | 1,000 |  |  |  |  |  |  |
| **INP** GS | 0,817 | **0,948** | 0,854 | 0,705 | 0,567 | 0,838 | 0,783 | 0,686 | 0,770 | 0,752 | 1,000 |  |  |  |  |  |
| **INP** IM | 0,781 | 0,817 | **0,954** | 0,641 | 0,522 | 0,780 | 0,728 | 0,675 | 0,736 | 0,720 | 0,763 | 1,000 |  |  |  |  |
| **OUT** GS | 0,737 | 0,784 | 0,833 | 0,740 | 0,636 | 0,822 | 0,786 | 0,672 | 0,727 | 0,732 | 0,718 | 0,736 | 1,000 |  |  |  |
| **OUT** IM | 0,691 | 0,726 | 0,740 | 0,702 | 0,536 | 0,697 | 0,731 | 0,655 | 0,697 | 0,685 | 0,732 | 0,700 | 0,721 | 1,000 |  |  |
| **SUR** GS | 0,811 | 0,795 | 0,775 | 0,774 | 0,664 | 0,829 | 0,761 | 0,722 | 0,753 | 0,763 | 0,786 | 0,727 | 0,768 | 0,736 | 1,000 |  |
| **DIA** AP | 0,465 | 0,431 | 0,417 | 0,517 | 0,575 | 0,495 | 0,479 | 0,492 | 0,477 | 0,514 | 0,407 | 0,390 | 0,435 | 0,320 | 0,448 | 1,000 |

Notes. BEDS, number of beds; ROO, number of rooms; PHY, number of physicians; RES, number of residents; NUR, number of nurses; OTH, other staff; INP, number of inpatient discharges; OUT, number of outpatient visits; SUR, number of performed surgeries; DIA, number of diagnostic and therapeutic procedures accomplished; GS, General Surgery; IM, Internal Medicine; AP, Anatomical Pathology.

**Table S3**- Fixed effects estimates

| Year | Fixed Effects | | |
| --- | --- | --- | --- |
|  | **General surgery** |  | **Internal Medicine** |
| 1999 | 0,1548 |  | 0,0904 |
| 2000 | 0,1413 |  | 0,0719 |
| 2001 | 0,0960 |  | 0,0740 |
| 2002 | 0,0759 |  | 0,0129 |
| 2003 | 0,0053 |  | 0,0056 |
| 2004 | -0,0121 |  | 0,0074 |
| 2005 | -0,0576 |  | -0,0352 |
| 2006 | -0,0604 |  | -0,0489 |
| 2007 | -0,1014 |  | -0,0552 |
| 2008 | -0,0970 |  | -0,0630 |
| 2009 | -0,1550 |  | -0,0658 |
| 2010 | -0,0803 |  | -0,0416 |
